# The Role of Mesenchymal Stem Cells in the Treatment of Type 1 Diabetes

**DOI:** 10.7759/cureus.27337

**Published:** 2022-07-27

**Authors:** Maleesha Jayasinghe, Omesh Prathiraja, Prashan B Perera, Rahul Jena, Minollie Suzanne Silva, P.S.H. Weerawarna, Malay Singhal, Abdul Mueez Alam Kayani, Snigdha Karnakoti, Samiksha Jain

**Affiliations:** 1 Medicine, Nanjing Medical University, Galle, LKA; 2 Medicine and Surgery, Nanjing Medical University, Melbourne, AUS; 3 Internal Medicine, Nanjing Medical University, Colombo, LKA; 4 Medicine, Bharati Vidyapeeth Medical College/Bharati Hospital, Pune, IND; 5 Medicine and Surgery, Nanjing Medical University, Nanjing, CHN; 6 Internal Medicine, Jiangsu Province People's Hospital, 1st Affiliated Hospital to Nanjing Medical University, Nanjing, CHN; 7 Internal Medicine, Mahatma Gandhi Memorial Medical College, Indore, IND; 8 Medicine and Surgery, Allama Iqbal Medical College, Lahore, PAK; 9 Medicine, Malla Reddy Institute of Medical Sciences, Hyderabad, IND; 10 Medicine, Guntur Medical College, Guntur, IND

**Keywords:** diabetic nephropathy, pancreas, insulin, mesenchymal stem cells, stem cells, diabetes, type 1 diabetes

## Abstract

Type 1 diabetes (T1D) is a chronic disease characterized by inadequate or absent insulin production due to the autoimmune destruction of beta (β) cells in the pancreas. It was once called "juvenile diabetes" since the disease frequently occurs in children, but it can also develop in adults. According to the International Diabetes Federation, an estimated 700 million adults will suffer from diabetes by 2045. Although the exact cause of diabetes remains unknown, it is hypothesized that genetic factors, environmental factors, and exposure to certain viruses play a role in the development of T1D. To date, exogenous insulin is the most common treatment for T1D. However, it is not a cure for the disease. Islet cell transplantation and pancreatic transplantation are two additional treatments that have gained popularity in recent years, but their clinical application may be limited by the need for high doses of immunosuppressants, the rarity of human cadaveric islets, and the need for extensive surgery in pancreatic transplantation. Mesenchymal stem cells (MSCs) are a highly promising novel treatment for T1D and their discovery has advanced biological sciences by allowing for modification of cell fate and the development of higher-order cellular structures. They play an essential role in lowering levels of fasting blood sugar, hemoglobin A1c, and C-peptide, and in treating microvascular complications associated with T1D. However, some of the disadvantages of its use in clinical practice are limited to its method of collection, proliferation rate, cell activity with age, and the risk of tumour formation identified in some studies. Large-scale studies are required to discover the mechanism of action of MSCs after administration as well as the optimal route, dose, and timing to maximize the benefits to patients. This article focuses primarily on the role of MSCs in the treatment of T1D and compares the feasibility, benefits, and drawbacks of MSCs in the treatment of T1D.

## Introduction and background

Type 1 diabetes (T1D) is a chronic immune-mediated disease characterized by the destruction of pancreatic β-cells, resulting in absolute insulin deficiency and hyperglycemia. It is primarily a disease of youth, accounting for approximately 85% of cases in people under the age of 20 and 5% to 10% of all diagnosed cases of diabetes [1,2]. Although the exact mechanisms are unknown, T1D is thought to develop through immune system activation against β-cell antigens and the initiation of proinflammatory cytokine responses. Environmental factors, obesity, viral infections, and nutritional factors were found to play a role in the pathophysiology as well [3]. T1D predisposes to a number of comorbidities, such as obesity, chronic kidney disease, metabolic syndrome, coronary artery disease, and hypertension. Such predispositions may account for higher mortality rates, affecting up to one in 10 adult patients within a year of diagnosis [4]. In fact, diabetic nephropathy (DN) is said to account for up to 40% of end-stage renal disease (ESRD) cases worldwide. Cardiovascular events account for up to 70% of T1D deaths and are 10 times more common in diabetics than in non-diabetics [5]. Therefore, it is critical to focus on novel therapies that aim to reduce the risks of acute complications such as hypoglycemia and diabetic ketoacidosis (DKA) while avoiding long-term complications such as DN, neuropathy, and retinopathy [5].

Exogenous insulin is currently the most prevalent treatment for T1D. Although exogenous insulin administration may be life-saving, it is not a cure for the disease. If patients are unable to maintain tight glycemic control by strictly adhering to their insulin regimen, they will invariably develop severe secondary complications that may shorten their life span [6]. Exogenous insulin is not a viable substitute for normal pancreatic islet function, mainly due to the absence of accurate temporal glucose control over time [7]. The administration of insulin can also result in hypoglycemic episodes [6]. A cross-sectional study conducted in Mexico revealed that patients' fear of hypoglycemic episodes prevented them from complying with their insulin treatment plan [8]. 

Replacement of the defective insulin-producing cells (IPC) is yet another potential therapy for T1D. This is possible through transplantation of the pancreas. Since the first pancreatic transplant took place in 1966, over 50,000 such transplants have been performed worldwide. Patients with T1D who receive a pancreatic transplant were found to have a reduced risk of subsequent complications and a longer life expectancy [9]. However, since transplantation is a major surgical procedure, patients must be fit for surgery [6]. Transplants necessitate permanent immunosuppression, which may put patients at risk for a variety of infections. In addition, they are associated with a number of postoperative complications, such as pancreatitis, due to low tolerance to cold ischemia, bleeding, thrombosis, and anastomotic leakage, which may require relaparotomy and graft pancreatectomy in recipients [9].

An alternative to pancreatic transplantation that is both safe and effective is islet cell transplantation. Scharp et al. published the first case of allogeneic intraportal islet transplantation for T1D in 1990, which led to short-term insulin independence and paved the way for clinical islet transplantation [10]. Despite the fact that the immunosuppressive regimen reported from Edmonton, Canada, also known as the Edmonton protocol (the Edmonton protocol introduced significant adjustments to the transplantation procedure, including the use of an immunosuppressive regimen free of steroids and the transplanting of an average islet mass of 11,000 islet equivalents per kilogram) has achieved unprecedented success in islet transplantation in terms of insulin independence, a number of factors continue to influence the outcome of this minimally invasive procedure [11]. Islet cell transplantation can induce a rapid inflammatory reaction in the circulation, leading to the loss of the vast majority of transplanted islets. The use of large doses of immunosuppressants during transplantation compromises the long-term viability and function of the graft, and the need for long-term immunosuppressive medications after the transplant poses a risk of organ damage, malignancies, new infections, and new-onset T1D in patients [12]. The high cost of islet transplantation and the paucity of human cadaveric islets highlight the urgent need for innovative pancreatic islet transplantation procedures [7]. This is where stem cells (SCs) pose an important role.

**Figure 1:**
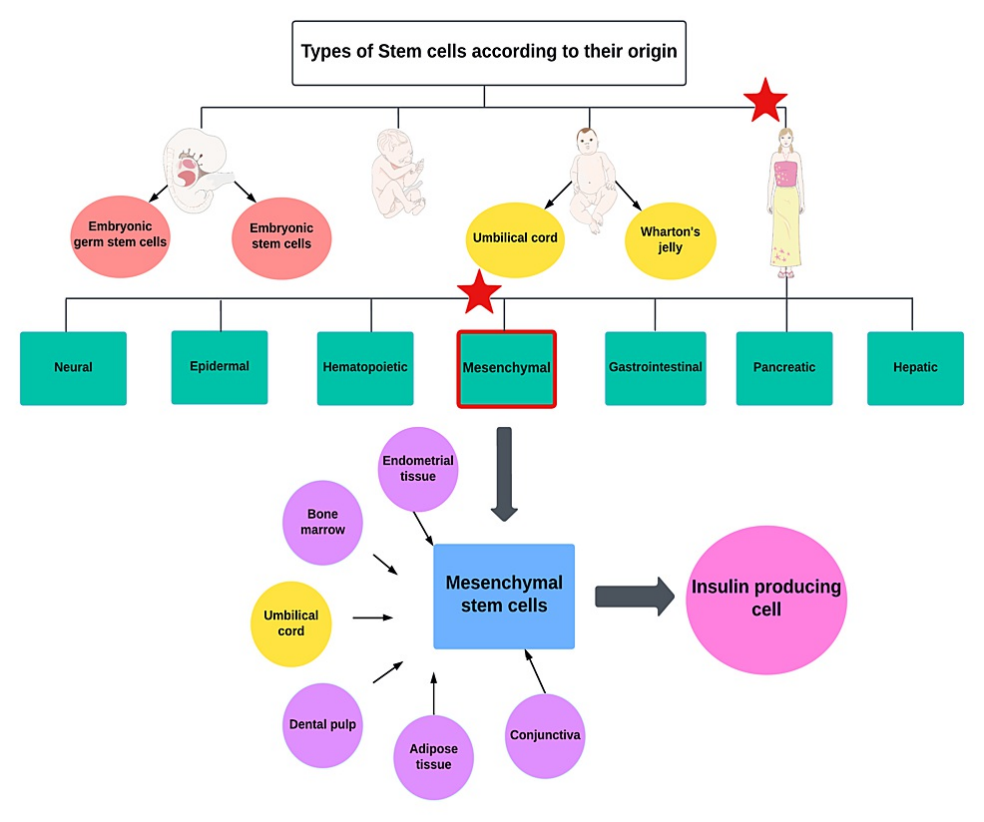
Classification of stem cells based on their origin Original figure, made by author Maleesha Jayasinghe The figure was partly generated using Servier Medical ART, provided by Servier (Les Laboratoires Servier, SAS, Suresnes, France), licensed under a Creative Commons Attribution 3.0 unported license.

SCs are a highly promising novel treatment for T1D due to their ability to differentiate into several cell types and their regenerative potential. SCs can be categorized into four basic groups based on their origin as shown in Figure 1.

Mesenchymal stem cells (MSCs), also called mesenchymal stromal cells, are non-hematopoietic, multipotent SCs. They can be extracted from a variety of sources, including bone marrow, liver, kidney, adipose tissue, urine, umbilical cord blood, umbilical tissue, Wharton's jelly, placenta, and even endometrial tissue (menstrual blood-derived endometrial stem cells - MenSC). Several surface markers, including CD73, CD90, and CD105, can be utilized to identify MSCs. Due to their ability to differentiate into numerous cell types, they can be used to repopulate damaged tissues [13,14]. MSCs have gained enormous popularity in the treatment of T1D because of their ability to regulate fibrosis and tissue regeneration, as well as their ability to modulate immunological function. In addition, they produce a variety of secretory molecules, such as cytokines and exosomes, which play an essential role in the treatment of T1D [15]. Studies on animals treated with MSCs have shown a significant reduction in hyperglycemia, as evaluated by a decrease in serum glucose and an increase in insulin and C-peptide levels. In addition, they were able to restore normal levels of lipid fractions. Using MSCs lowered the serum levels of both liver and kidney function markers in diabetic rats, demonstrating their hepato-renal protective benefits in T1D [16].

**Figure 2:**
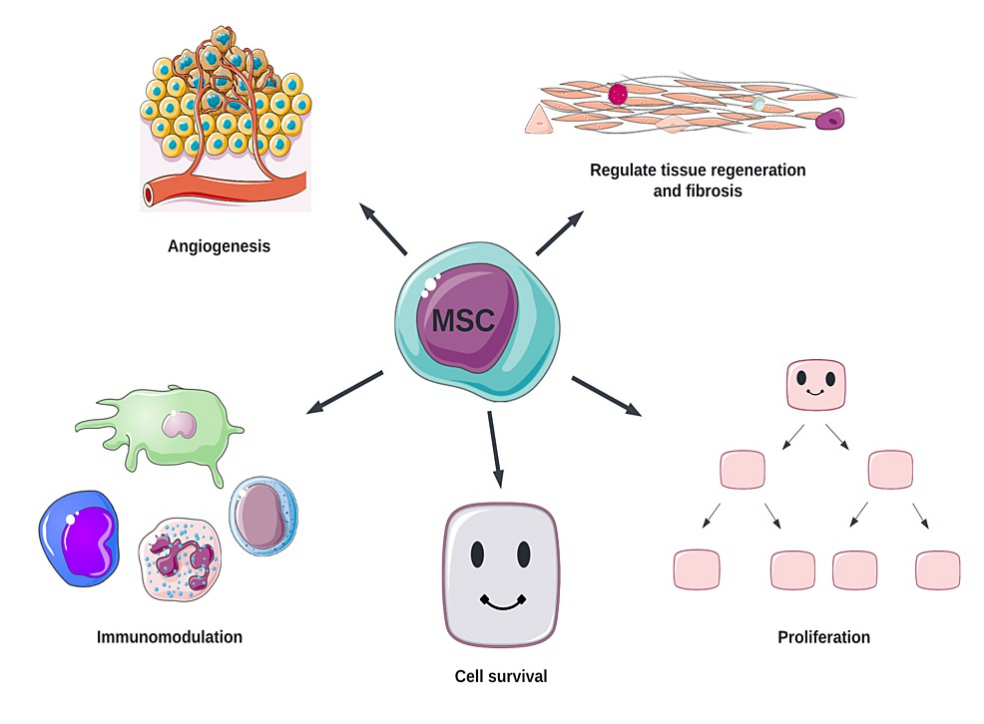
Mechanisms of action of MSCs MSC: mesenchymal stem cell Original figure, made by author Maleesha Jayasinghe The Figure was partly generated using Servier Medical Art, provided by Servier, licensed under a Creative Commons Attribution 3.0 unported license

Several mechanisms have been discovered to play a role in the management of T1D by MSCs (Figure 2).

MSCs, such as bone marrow stromal cells, promote angiogenesis through the secretion of cytokines such as basic fibroblast growth factor and vascular endothelial growth factor (VEGF) [17]. In addition, they play a crucial role in immunomodulation by moving to areas of inflammation and modifying the phenotype of dendritic cells (DC), T cells, B cells, and natural killer cells. They downregulate proinflammatory cytokines and escape CD8+ T cell-mediated apoptosis, inhibit maturation of DC, while reducing T-lymphocyte proliferation via transforming growth factor-beta 1 (TGF-β1), hepatocyte growth factor, and nitric oxide. By stimulating the production of regulatory T cells, TGF-β1 plays a significant role in the immunomodulation of MSCs. MSCs have also been found to improve the function, survival, and graft outcome of neonatal porcine islets by increasing the expression of genes involved in the formation of endocrine cells, insulin, and platelet-derived growth factor alpha (PDGFR-α). PDGFR-α suppresses Notch 1 signaling (Notch 1 downregulates transcription factors involved in the formation of endocrine cells and insulin), resulting in the maturation and development of islet cells [18]. Zhou et al. discovered that wild-type p53-induced phosphatase 1 (a serine/threonine phosphatase) regulates the immunomodulatory properties of MSCs via the expression of interferon-alpha and bone marrow stromal cell antigen 2, consequently playing an important role in the therapeutic effects of MSCs in T1D [19]. 

Even though studies have shown that MSCs are capable of reconfiguring the immune system, they must be rescued to some extent from immune-mediated destruction, indicating that immunomodulation will be necessary even if a viable MSCs therapy for T1D is produced [20]. When using β-cells from an allogeneic stem cell source, an alloreactive response to donor antigens will be generated unless we obtain SCs from the patient's own cells. To circumvent this, researchers have investigated encapsulation strategies employing semipermeable immune barriers to provide immune shielding and prevent graft rejection [21]. Some studies have also demonstrated that the use of suicide genes together with stem cell transplants promotes functional immune reconstitution and thereby prevents graft-versus-host disease in patients [22]. 

It has been demonstrated that MSCs undergo apoptosis in the circulation of the host or in engrafted tissues following delivery to the patient's body, which plays a significant part in their therapeutic role in T1D. During the execution of apoptosis, apoptotic extracellular vesicles (apoEVs), formerly known as apoptotic bodies, have emerged as regulators of numerous biological processes, as opposed to being only debris. Specifically, apoEVs have been shown to regulate T cell and macrophage immunological function as well as stimulate tissue repair, including skin regeneration and vascular protection [23]. 

This game-changing discovery of MSCs in the treatment of T1D has propelled biological sciences to a new level of sophistication, allowing for the manipulation of cell fate and the cultivation of higher-order cellular structures. However, there is still a huge gap regarding its application in actual clinical practice.

We were only able to find 12 clinical trials on PubMed that evaluated the use of MSCs in the treatment of T1D. Ten of the 12 studies were undertaken in Asia, primarily in China and India. To date, the exact pathogenesis of T1D is not fully understood. Genetic factors have been found to play a role in the development of T1D, which may have affected the outcomes of previous clinical trials. Therefore, conducting multiple different studies worldwide would not only enable us to identify the effects of ethnicity and genetics on the response to MSC therapy in T1D patients but also help us to generalize the efficacy of MSCs to the entire population. In order to achieve the best outcomes while using medications to treat T1D, it is also crucial to perform additional research to more clearly identify the pathophysiology of T1D. 

In the course of studying the patient selection criteria utilized in clinical trials, we made a fascinating discovery. We found that every clinical study had excluded patients with immunosuppression, viral illnesses such as hepatitis B and C, comorbidities including hematologic diseases, rheumatologic diseases, and kidney diseases, and pregnant patients, all of which could have influenced the results of the studies. Our present understanding of the action of apoEVs, as described by Fu et al., leads us to believe that in order for MSCs to undergo apoptosis, their recipients must be able to initiate apoptotic activity [23]. In order for this to occur, patients must have a particular number of cytotoxic T cells or natural killer cells; hence, patients who do not meet this criterion are unlikely to benefit from MSC delivery. To further elucidate the mechanisms of action of MSCs, it is essential to undertake additional studies with immunosuppressed patients in order to identify the optimal cohort of T1D patients for MSC therapy. In addition, further clinical research should be conducted to uncover the apoptotic signals that stimulate tissue regeneration and angiogenesis, as recognizing these signals would allow us to utilize a channel in parenchymal tissue to increase its regeneration capacity. 

We also observed that the majority of trials exclusively enrolled patients with recent-onset T1D. A study conducted in Iran revealed that early transplantation of MSCs resulted in superior outcomes for T1D patients compared to late transplantation. This may be due to the honeymoon phase of diabetes, which may have obscured the effects of MSCs in these studies [24]. The honeymoon phase is the period during which a person with T1D appears to improve and may only require minimal amounts of insulin or experience normal or near-normal blood sugar levels without insulin. To extrapolate the results to a larger population and unmask the effects of the honeymoon period, it is necessary to conduct trials on patients with late-onset T1D. 

To date, the exact mechanism by which MSCs contribute to the remission of T1D has not been identified; therefore, further research is required to get a better knowledge of mechanisms such as immunomodulation, homing, and paracrine signaling of MSCs. It is also vital to undertake studies to discover the appropriate number of MSCs, injection frequency, and optimal infusion route in order to maximize results. Cai et al. concluded that pancreatic arterial transfusion would assist in avoiding the first pass pulmonary effect of MSCs, hence lowering the sequestration of MSCs in the lungs and allowing for optimal results [25]. 

A few studies have used 3D microspheres to increase the proliferation capacity of MSCs with positive results. However, there is insufficient information available regarding the proliferation capacity, revascularization, efficiency of differentiation, and survival time of MSCs. Therefore, conducting studies to elucidate these aspects of MSC therapy is an urgent necessity. We would also be able to learn more about the graft's survival time and tumorigenic potential if we followed the patients for a longer period of time. 

Patient-specific variables such as age, body mass index, lifestyle, socioeconomic status, level of activity, diet, autoimmune status, and drug interactions must be taken into consideration while conducting studies and analyzing data. In order to identify the ideal conditions necessary to create the desired quantities of MSCs to achieve remission of T1D, future research must also incorporate in-depth information regarding external factors that affect the viability of MSCs, such as storage conditions, plating density, and culture media.

In this article, we aim to discuss the role of MSCs derived from various tissues in the treatment of T1D, as well as their feasibility and limitations.

## Review

We present a summary of the extraction methods, advantages, limitations, and outcomes from several studies of MSCs derived from various types of tissues.

Umbilical cord tissue-derived mesenchymal stem cells 

The majority of umbilical cord tissue-derived stem cells (UC-MSCs) are found in the subcortical endothelium of the umbilical cord, the perivascular area, and Wharton's jelly [26]. According to studies, roughly 1 × 10^6^ UC-MSC can be extracted from a 20 cm human umbilical cord [27]. MSCs isolated from Wharton's jelly have been grown for over 80 population doublings without showing any signs of senescence, morphological alterations, an increase in growth rate, or a change in their ability to develop into neurons. Recent research has demonstrated that xenotransplantation of post-differentiated human UC-MSC without immunosuppressive therapy does not result in rejection [28]. This lack of immunogenicity may be attributable to the absence of major histocompatibility II and co-stimulatory molecules such as CD80 (B7-1), CD86 (B7-2), and CD40 [29]. Chao et al. successfully differentiated human UC-MSC into clusters of mature islet-like cells with insulin-producing capacity. In the islet cells, they detected an increase in insulin and other β-cell-related genes, including *Pdx1*, *Hlxb9*, *Nkx2.2*, *Nkx6.1*, and *Glut-2*. Moreover, they discovered that hyperglycemia in diabetic rats was greatly under control after xenotransplantation of human pancreatic islet-like cell clusters [28]. Patients with newly diagnosed T1D who received repeated intravenous doses of allogeneic UC-MSC showed improved islet cell preservation and a significant rise in postprandial C-peptide levels. However, C-peptide levels did not alter significantly in patients with juvenile-onset T1D. The number of UC-MSC contributed more than other indicators to the prediction of clinical remission, bolstering the evidence of dose-dependent therapeutic efficacy. Therefore, appropriate doses and courses of MSC transplantation should be granted importance in future research [30].

UC-MSC can also be used to treat chronic complications of T1D, such as neuropathy, DN, and retinopathy [31]. Studies have shown that intraperitoneal injection of human UC-MSC can ameliorate renal injury in streptozotocin-induced diabetic mice. [32]. A mice study conducted in China demonstrated that the combination of human UC-MSC and resveratrol can better protect renal podocyte function and the resulting reduction in blood glucose levels and renal damage is superior to those obtained with insulin administration [33]. This suggests that the combination of resveratrol and human UC-MSC may be an innovative technique for treating T1D; however, additional research on humans is necessary to determine the effects of this combination treatment on the management of DN. Another investigation involving mice revealed that UC-MSC therapy restored erectile function by suppressing toll-like receptor 4, alleviating corpora cavernosa fibrosis, and boosting the production of VEGF and endothelial nitric oxide synthase [34]. Nonetheless, a significant advantage of UC-MSC is that they are a rich source of many SCs that can be easily manipulated [27]. They are collected at delivery by clamping and severing the umbilical cord. There are no ethical concerns regarding the use of UC-MSC because the collecting process is non-invasive and retains material that would otherwise be discarded as waste.

Adipose tissue-derived mesenchymal stem cells 

Adipose tissue-derived mesenchymal stem cells (ADSCs) are a group of cells that arise from the mesoderm during embryonic development. Amongst several types, subcutaneous adipose tissue seems to be the most clinically relevant source, being available in abundance for harvest, and its isolation only slightly invasive [35,36].

While two major kinds of adipose tissue (white and brown) have been isolated and studied, we focus on white adipose, which produces ADSCs, as brown adipocytes have not yet demonstrated an association with insulin resistance. White adipose tissue expressing uncoupling protein 2 (an isoform of uncoupling protein 1 in brown adipose) acts as a storage of excess energy in the form of triglycerides and is thus prone to causing obesity and abnormalities in metabolic pathways such as insulin resistance during hyperplasia [37].

The extracted cell group of interest consists of a putative stem cell population of fibroblast-like cells known as processed lipoaspirate (PLA), found within the stromal compartments of adipose tissue [38]. Obtaining the sample requires lipoaspiration, and although the technique does not negatively affect the function of ADSCs, the vacuum process involved can cause damage to mature adipocytes [37]. Studies have shown that successfully extracted PLA can then differentiate *in vitro* into multiple cell lineages (including adipogenic, myogenic, chondrogenic, and osteogenic cells), thus providing another source of SCs with multi-germ-line potential instead of the traditional bone marrow-derived MSCs [38-41]. The discovery of the ability of ADSCs to efficiently differentiate into IPC has shed new light on the approach to T1D management [41]. 

ADSCs utilization can help avoid ethical barriers and tumorigenic complications that are increasingly encountered during stem cell isolation from embryos and induced pluripotent SCs [36]. Yet another advantage of ADSCs for their therapeutic application happens to be the relatively painless procedure and high yields in harvested cell numbers compared to bone marrow procurement [40]. These cells are devoid of human leukocyte antigen-DR expression and therefore have been successfully transplanted via intravenous, intraperitoneal, and renal capsule administration in mice without the need for immunosuppression [36,42].

Insulin replacement therapy with the help of co-transplantation of insulin-secreting ADSCs has been studied as an alternative to lifelong insulin therapy. As with multiple studies, no adverse effects were observed with ADSCs infusion, and in fact, an impressive absence of DKA episodes in all participants was seen [43]. A prospective study conducted in 2015 on 20 patients with T1D found better diabetic control (hemoglobin A1c levels) and sustained improvements in fasting blood sugar, postprandial blood sugar, hemoglobin A1c, and C-peptide levels with the transplantation of autologous insulin-secreting ADSCs [44]. Dantas et al. concluded that combination therapy with ADSCs and Vitamin D (daily cholecalciferol for six months) without immunosuppression was safe, demonstrated immunomodulatory effects, and may play a role in β-cell preservation in patients with newly diagnosed T1D [45]. The significant functional and morphological improvements in islet cells as early as two months after transplantation of IPC clusters derived from ADSCs point to the promising nature of this therapeutic approach for achieving target normoglycemia [46,47]. A recent study conducted in 2022 discovered that systemic administration of ADSCs protects male non-obese diabetic (NOD) mice against diabetes induced by programmed death-1 and programmed death-ligand 1 (PD-1/PD-L1) inhibition. Multiple injections of neutralizing antibodies against mouse PD-L1 induce a significant infiltration of immune cells in the islets and a decrease in the β-cell area and insulin content of the pancreas. Despite this, systemic ADSC injection partially protected the pancreas from β-cell loss and preserved insulin content, indicating therapeutic potential in T1D [15]. 

Apart from the therapeutic uses in T1D, the ADSC therapy has also been shown to reduce adverse effects brought about by complications such as DN and ESRD [48,49]. Inactivation of nuclear factor kappa B pathways and downregulation of VEGF-A, amongst others, are the major mechanisms involved in ameliorating the pathological manifestations of mice with DN [50].

The problem remaining, however, is the inability to become totally free of exogenous insulin. Research suggests that a much larger dose of IPC may be required for a sustained cure of T1D using ADSCs [51]. Therefore, the need of the hour is to conduct further research, placing emphasis on ways to either enhance the production of insulin in IPC derived from ADSCs or alter cell signaling pathways to obtain a greater number of IPC from ADSCs. 

Bone marrow-derived mesenchymal stem cells

Bone marrow-derived mesenchymal stem cells (BM-MSCs) are a type of adult stem cell that is abundant in bone marrow and has low immunogenicity [52]. Bone marrow stem cells are broadly categorized into hematopoietic stem cells and MSCs. These cells are sourced from the same individual, potentially minimizing rejection problems and making it a form of therapy for T1D [53]. BM-MSCs can differentiate into functionally competent β-cells *in vivo*, and NOD mouse studies have shown the formation of normal T cell and B cell function, implying that allogeneic bone marrow transplant could prevent islet destruction and restore self-tolerance [54,55]. Because of their well-documented hypoimmunogenic and immunomodulatory properties, BM-MSCs are an appealing therapeutic option for T1D [56].

One study looked at T1D patients with DKA and found BM-MSCs to preserve β-cell function in T1D patients, reducing levels of fasting and post-prandial C-peptide levels, with one patient achieving insulin independence for a period of three months [57].

BM-MSCs have been demonstrated to mitigate the effects of metabolic and hepato-renal abnormalities, enhance lipid profiles, and improve carbohydrate and glycemic management. Following an eight-week period of injections with BM-MSCs in diabetic rats, an improvement was observed in their lipid profiles compared to diabetic rats that were not treated with BM-MSCs [16]. In addition, BM-MSCs therapy has been demonstrated to ameliorate diabetes-related liver damage by boosting endogenous hepatocyte regenerative mechanisms and enhancing liver function [58]. 

BM-MSCs have also been shown to effectively treat comorbidities of T1D, such as DN, poor wound healing, and erectile dysfunction (ED). Nagaishi et al. investigated a novel approach of mixing BM-MSCs with umbilical cord extracts in Wharton's Jelly to enhance the therapeutic effect of ameliorating renal injury in T1D patients with DN. The study demonstrated morphological and functional improvements of diabetes-derived BM-MSC *in vitro* and a therapeutic impact on DN *in vivo*, suggesting that this may be beneficial not only for patients with DN but also for patients with other diabetic complications [59]. One study looked to address the problem of impaired epithelial wound healing in T1D patients and found that BM-MSCs promote corneal epithelial wound healing via tumor necrosis factor-inducible gene 6-dependent stem cell activation [60]. Another promising phase I pilot clinical trial found that treating ED in T1D patients with two consecutive intracavernous injections of autologous BM-MSC was safe and effective [61].

Currently, several potential therapeutic approaches are being postulated to approach this issue of T1D from a new viewpoint. Suicide gene therapy is a strategy with potential. This method involves the introduction of suicide-inducing transgenes into the body via BM-MSC. As a result, several processes will be induced, including the suppression of gene expression, the production of intracellular antibodies that block the essential pathways of cells, and the transgenic expression of caspases and deoxyribonucleases. Current clinical trials are examining strategies to restore damaged organs with the use of stem cells as the delivery mechanism [62]. 

The idea of transplanting BM-MSCs provides patients with hope. Particularly significant are autologous BM-MSC (which are easy to obtain and avoid graft rejection after transplantation) in contrast to allogeneic BM-MSC transplantations, which may result in graft rejection and be accompanied by complications [52]. For stem cell therapy to be most beneficial, early delivery of stem cells following a diagnosis of T1D is necessary compared to intervention at later stages [63].

Table 1 compares the properties of MSCs derived from the bone marrow, umbilical cord, and adipose tissue.

**Table 1:** A comparison of BM-MSCs, ADSCs and UC-MSCs BM-MSCs: bone marrow-derived mesenchymal stem cells; ADSCs: adipose tissue-derived mesenchymal stem cells; UC-MSCs: umbilcal cord-derived mesenchymal stem cells Original table, made by author Minollie Silva

Property	UC-MSCs	BM-MSCs	ADSCs
Proliferation rate	Medium	Higher than ADSCs	Lower than BM-MSCs
Tissue processing and culture of cells	Easy	Easy	Easy
Harvesting technique	Non-invasive	Invasive	Invasive
Effect of donor age on cells	Unaffected	Decline with age	Decline with age
Cellular rejection	Can not be seen	Can not be seen	Can not be seen
Tumor formation risk	Low	Low	Low
Properties of anti-inflammation	Good	Good	Good
Expression of embryonic markers	High	Low	Low

Endometrium, dental pulp and conjunctival tissue-derived mesenchymal stem cells

Recent research has demonstrated that menstrual blood-derived endometrial stem cells (MenSCs) have therapeutic promise for the treatment of T1D due to their exceptionally high rates of proliferation, noninvasive collection method, and significant immunomodulatory activity. In T1D model mice, MenSC and UC-MSC transplantation resulted in a significant decrease in blood glucose and insulin levels, as well as an improvement in the morphology and function of the liver, kidneys, and spleen [14]. A 2021 study found that MenSCs expressed pancreatic β-cell genes such as *INSULIN*, *GLUT-2*, and *NGN-3* and had a greater capacity to develop into pancreatic cells [64].

Dental pulp-derived mesenchymal stem cells (DP-MSCs) are one of the unique MSCs proposed for the treatment of T1D. DP-MSCs are derived from exfoliated human deciduous teeth and have the properties of being easy to obtain with minimal donor injury. In a study by Mo et al. DP-MSCs revealed the ability to differentiate into pancreatic β-cells; nevertheless, before proceeding with larger-scale investigations to firmly establish this approach, it is necessary to devise procedures for optimal β-cell differentiation *in-vivo* [65].

An *in-vivo* study revealed that conjunctiva-derived mesenchymal stem cells (C-MSCs) efficiently differentiated into pancreatic islet stem cells in 2D cultures and 3D scaffolds under optimal induction conditions. C-MSCs have a strong proliferative capacity, a spindle shape, a high potential for clonogenic differentiation, and are widely available. However, larger *in vitro* studies are necessary before C-MSCs can be deemed an established treatment for T1D [64].

Table 2 lists all clinical trials that have utilized MSCs in the treatment of T1D and complications related to T1D (Table 2).

**Table 2:** A compilation of all the clinical trials on the response of T1D patients to MSCs therapy. ICA: islet cell antibody; GAD: glutamic acid decarboxylase; IA-2A: islet antigen-2 autoantibody; IL-4: interleukin-4; IL-6: interleukin-6; TGF-β1: Transforming growth factor beta 1; TNF-α: tumor necrosis factor alpha; T1D: type 1 diabetes; HbA1c: hemoglobin A1c; FBS: fasting blood sugar; PPBS: post prandial blood sugar; ADSC: adipose-derived stem cells; IPC: insulin-producing cells; ED: erectile dysfunction

Author	Type of study	Sample size	Inclusion criteria	Results of study	Adverse effects	Study weaknesses
Izadi et al. [24]	A triple-blinded parallel randomized placebo-controlled trial	21	Fasting C-peptide level ≥ 0.3 nmol/L, presence of at least one of three autoantibodies against pancreatic β cells (ICA, GAD, or IA-2A)	The number of hypoglycemic episodes and HbA1c levels was significantly reduced, with an increase in IL-4, IL-10, and TGF-β1 and a decrease in TNF-α, IL-6, and other pro-inflammatory cytokines.	Mild injection site reaction, urticaria, and a mild increase in lymphocytes.	Only a limited number of patients met the defined eligibility criteria, which caused a longer than expected recruitment process. Patient-specific variables such as lifestyle, socioeconomic status, stress level, exercise, and diet were not considered. All participants were enrolled in their early stages of diagnosis of T1D.
Cai et al. [25].	A Pilot Randomized Controlled Open-Label Clinical Study	42	Age 18–40 years, history of T1D ≥2 years and ≤16 years, HbA1c ≥7.5% (58 mmol/mol) and ≤10.5% (91 mmol/mol), fasting serum C-peptide <0.1 pmol/mL, and daily insulin requirements <100 IU	There was an increase in the C-peptide area under the curve and insulin area under the curve, and a reduction in HbA1c, fasting glycemia, and daily insulin requirements.	Severe hypoglycemic events, transient abdominal pain, and upper respiratory tract infections.	Relatively small sample size and a short duration of follow-up. The independent contribution of each cell product was not assessed separately. Insulin independence was not achieved. The lack of a placebo may generate bias in the quality of life measurements, which should be verified in a future large-scale study.
Lu et al. [30].	A non-randomized, open-label, parallel-armed prospective study	52	Age eight to 55 years (insulin requirement since diagnosis of T1D) and a fasting C-peptide level ≥ 100 pmol/L	There was a 10% increase in the level of fasting and/or postprandial C-peptide from baseline in 40.7% of patients. Three subjects achieved insulin independence and remained insulin-free for three to 12 months. The percent change of postprandial C-peptide was significantly increased in patients with adult-onset T1D. Changes in fasting or postprandial C-peptide were not significant among patients with juvenile-onset T1D.	Mild fever	A disparity in insulin requirements between the two groups at baseline suggests the possibility of non-randomized design-related selection bias. Due to the excessive length of the experiment, potentially confounding variables were introduced during data collecting.
Thakkar et al. [44].	A prospective, open-labeled, two-armed clinical trial.	20	T1DM of >12 months duration, presence of GAD antibodies, age eight to 45 years, low C-peptide levels	Sustained improvement in HbA1c, C-peptide, mean FBS, and PPBS. Reduction in GAD antibodies and insulin requirements.	Not reported	Insulin independence was not achieved.
Dantas et al. [45].	A prospective, dual-center, open trial	17	American Diabetes Association criteria for < 4 months, age 16 to 35 years, and positive GAD antibodies.	An increase in basal C-peptide levels after six months. C-peptide level and area under the curve for c-peptide remained stable for six months.	Transient headache, mild local infusion reactions, tachycardia, abdominal cramps, local thrombophlebitis, transient mild eye floaters during infusion, and central retinal vein occlusion (with complete resolution).	The study included only a limited number of patients. It was not possible to determine whether the beneficial effect of ADSC in pancreatic function was due to immune modulation or secondary to their differentiation in beta cells. This was an open study, and most participants accepted entry only to the intervention arm.
Al Demour et al. [61].	A prospective phase 1 pilot, open label, single arm and single center study.	4	Adult male patients, age 25 to 65 years, Type 1 or Type 2 diabetes, history of diabetes ≥5 years, HbA1c ≤10%, history of chronic ED for at least six months, body mass index between 20 and 30, and a baseline International Index of Erectile Function (IIEF-15) score of <26. Only patients with proven unresponsiveness to previous medical therapies such as PDE5 inhibitors and prostaglandin E1 were considered.	Significant improvement in the International Index of Erectile Function-15, Erection Hardness Score, sexual desire, intercourse satisfaction, and overall satisfaction.	None	The study included a limited number of patients due to the low social acceptance of this new treatment modality.
Carlsson et al. [66].	Open single-center randomized pilot study	20	Age 18-40 years of age and new-onset T1D.	The control arm showed a loss in both C-peptide peak values and C-peptide under the curve during the first year.	Viral upper respiratory tract infections, microscopic colitis, and Horton’s headache	There was a greater number of females than males in the control group. In larger studies, however, no effect of gender on the depletion of C-peptide has been documented.
Araujo et al. [67].	A prospective, single-center, open trial	13	American Diabetes Association criteria for < 4 months, age between 16 and 35 years, and presence of GAD antibodies.	Better glycemic control and lower insulin requirements were observed. Neither basal C-peptide nor age was associated with a decrease in HbA1c. There was an improvement in the HbA1c level and a higher frequency of CD8+FoxP3+ T cells.	Transient headache, mild local infusion reactions, tachycardia, abdominal cramps, local superficial thrombophlebitis, transient mild eye floaters during infusion, and central retinal vein occlusion	The study included a small sample size. There was a lower baseline C-peptide level in the control group, compared to the intervention group. There was no group treated solely with vitamin D and insulin to determine if the positive results were related to allogeneic ADSC, vitamin D, or both. A longer follow-up is necessary to determine the long-term safety and efficacy of this intervention. The study only selected T1D patients with fasting C-peptide ≥0.3ng/ mL.
Hu et al. [68].	A double blind study divided into two groups by randomized blocks	29	Age not exceeding 25 years, clinical and laboratory diagnosis of T1D according to the American Diabetes Association criteria, duration of T1D not more than 6 months, and fasting C-peptide ≥ 0.3 ng/mL.	Significant improvement in HbA1c and C-peptide levels.	None	The study included a limited number of patients. Only selected T1D patients with fasting C-peptide ≥0.3ng/ mL in the study.
Dave et al. [69].	Prospective non-randomized open-labeled clinical trial	10	Age eight to 45 years, confirmed diagnosis of TID for at least six months and low levels of C-peptide.	There was an improvement in mean Hb1Ac. An increase in mean serum C-peptide along with a decrease in exogenous insulin requirement was observed. There was a reduction in mean GAD antibodies.	None	Unanswered problems include the effects of immunological rejection on IPC, the dose of cells required to achieve complete treatment, and the engraftment technique or need for more potent cells like regulatory T cells.

Limitations

Our article relies on a survey of free full-text research journals over the past decade; consequently, it is possible that we have omitted pertinent information from paid full-text as well as research articles published prior to 2010. In addition, the scope of this study is confined to studies in the English language, so we may have overlooked papers published in other languages.

## Conclusions

MSCs are postulated to act in T1D and numerous other disorders through diverse mechanisms. Among these are homing and immunomodulation. Our review revealed that MSCs not only effectively reduce fasting blood sugar, C-peptide, and hemoglobin A1c levels but are also capable of treating microvascular complications associated with T1D. However, the specific pathophysiology of T1D diabetes is still unknown, making it difficult to develop novel treatments. To achieve remission of T1D, we must also consider the effects of additional factors on the efficacy of MSCs, including patient-specific variables such as age, body mass index, lifestyle, socioeconomic status, level of activity, diet, autoimmune status, and drug interactions, as well as external factors such as storage conditions, plating density, and culture media. Therefore, it is urgent to conduct larger-scale studies. 
